# Supplementary material for: Prediction of Parkinson’s Disease Risk Based on Genetic Profile and Established Risk Factors
Source: Genes (Basel). 2021 Aug 20;12(8):1278. doi: 10.3390/genes12081278 (PMC8393959; doi:10.3390/genes12081278)
Supplement: Supplementary file 1 [file genes-12-01278-s001.zip › Supplementary file legends.pdf]

Table S1: Demographic characteristics and lifestyle/environmental exposure risk factors of Cypriot PD cases and controls after imputation.

Table S2: Univariate logistic regression analysis of lifestyle/environmental risk factors after imputation.

Table S3: ORs, 95% CI and distribution of cases and controls in PRS quartiles after imputation.

Table S4: ORs, 95% CI and distribution of cases and controls in BMI quartiles after imputation.

Table S5: Stepwise-regression analysis by forward selection, backward elimination and bidirectional elimination approaches using the imputed data.

Table S6: ORs, 95%CI and distribution of cases and controls in deciles of the final multivariate-model after imputation.

Figure S1: PRS distribution between PD cases and controls after imputation. This plot shows the probability density versus PRS in cases and controls.

Figure S2: Cases and controls distribution in deciles using the multivariable model after imputation. The distribution of cases and controls are described in blue and orange, respectively.

Figure S3: OR by decile of the multivariable model after imputation.
