# Supplementary figures and images for: Prediction of Parkinson’s Disease Risk Based on Genetic Profile and Established Risk Factors
Source: Genes (Basel). 2021 Aug 20;12(8):1278. doi: 10.3390/genes12081278 (PMC8393959; doi:10.3390/genes12081278)

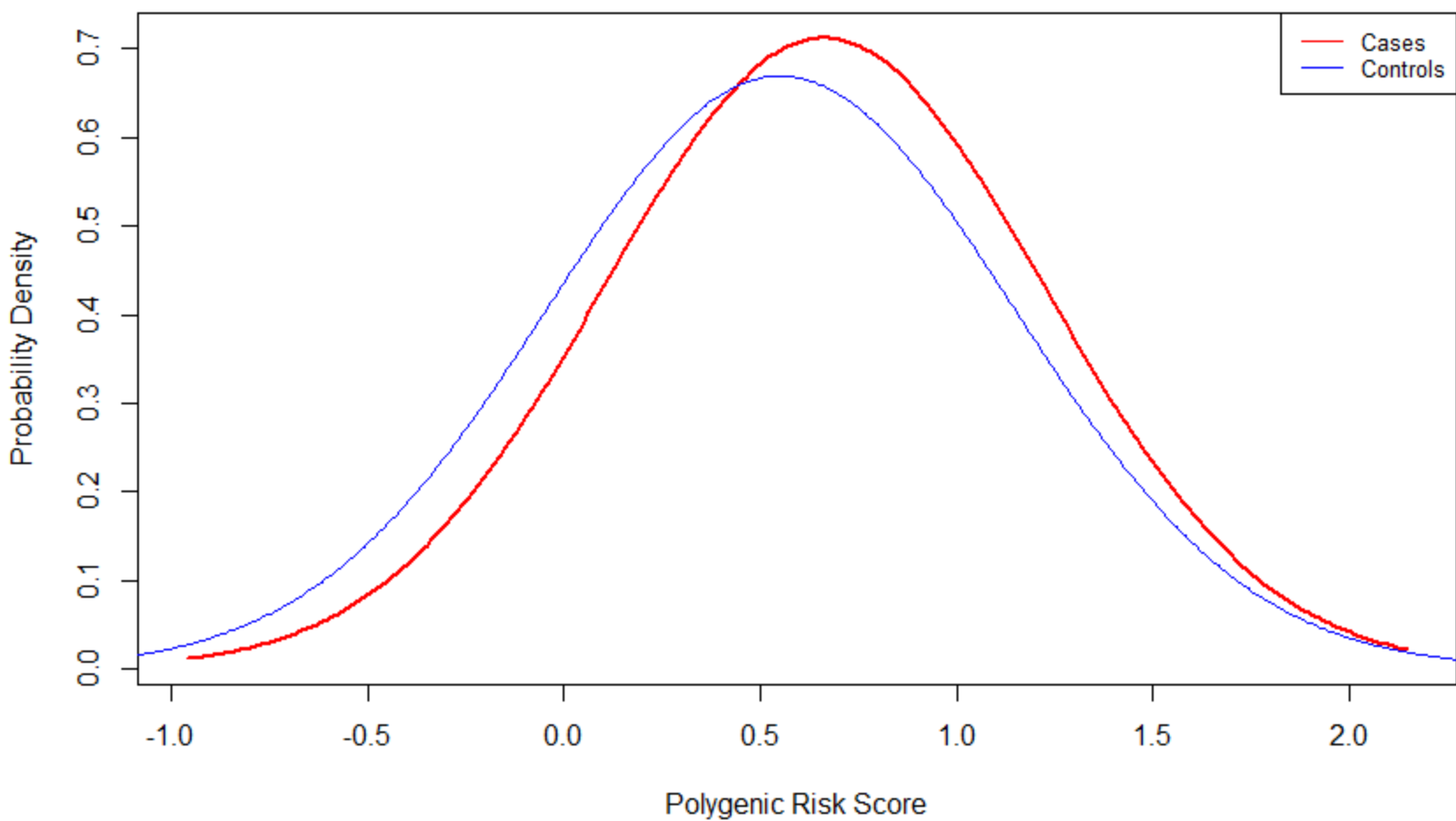

Supplement: Supplementary file 1 [file genes-12-01278-s001.zip › Figure S1.pdf]

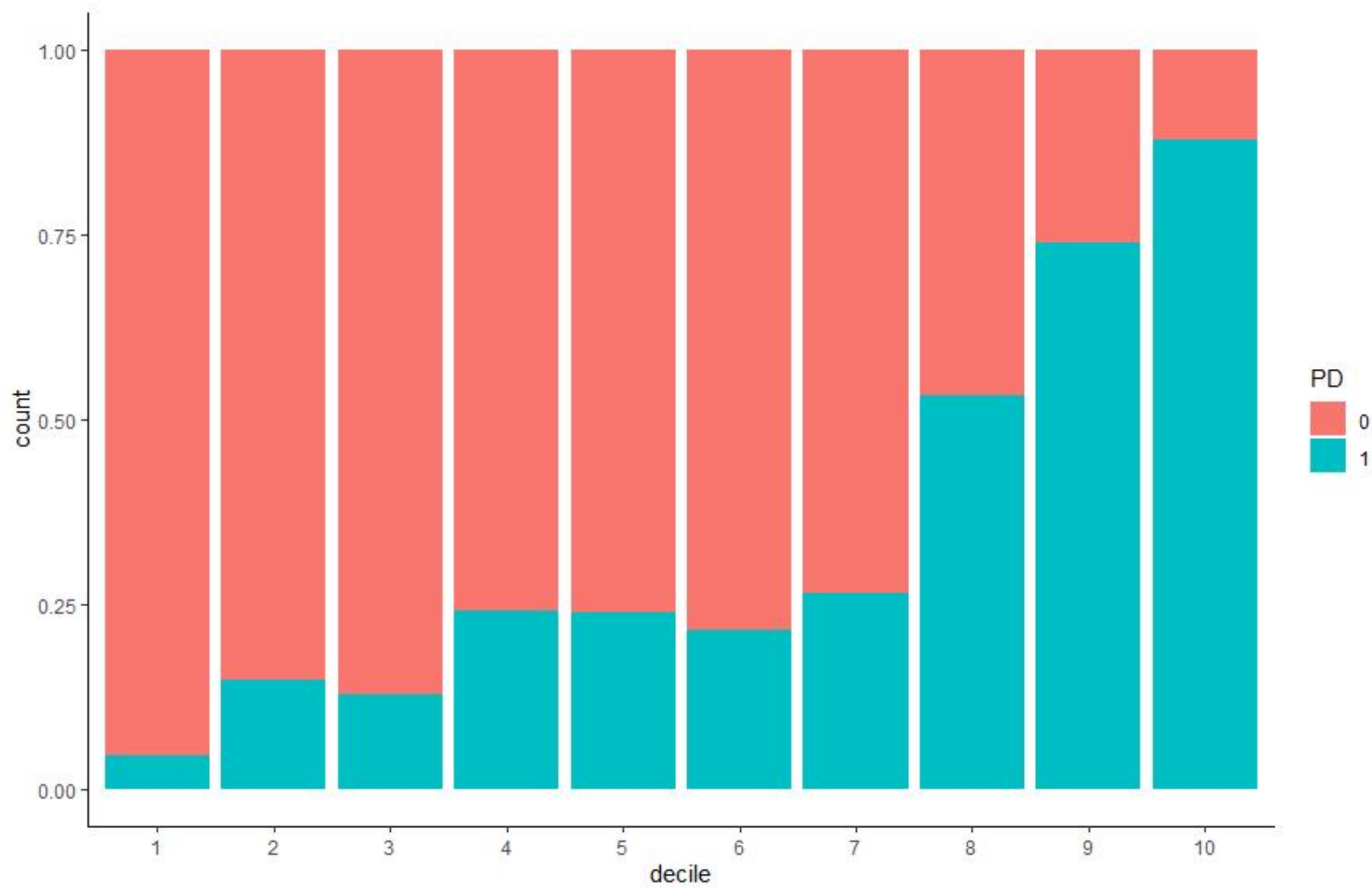

Supplement: Supplementary file 1 [file genes-12-01278-s001.zip › Figure S2.pdf]

Deciles

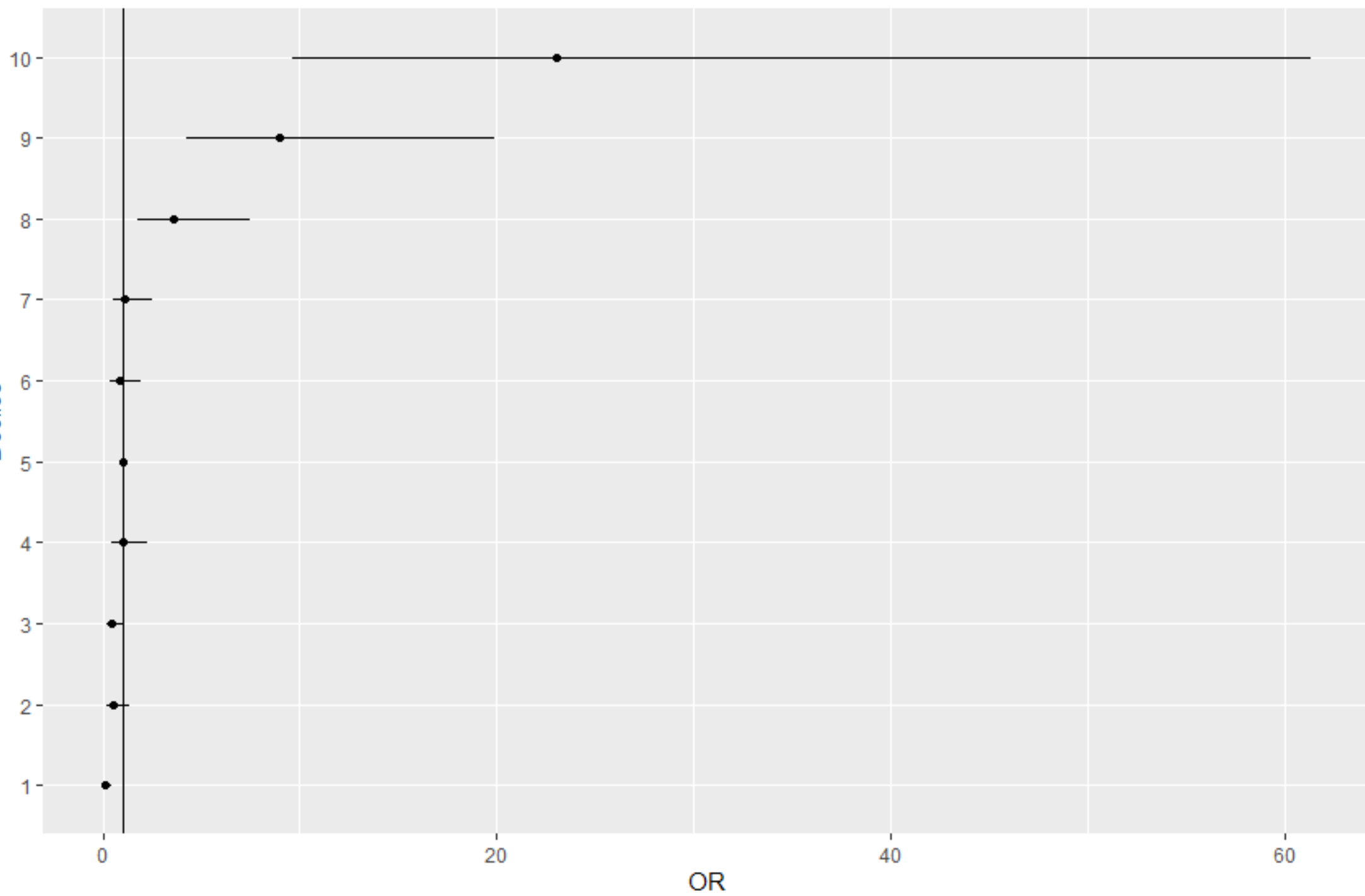

Supplement: Supplementary file 1 [file genes-12-01278-s001.zip › Figure S3.pdf]
